# Guiding longitudinal sampling in IBD cohorts

**DOI:** 10.1136/gutjnl-2017-315352

**Published:** 2017-10-21

**Authors:** Yoshiki Vázquez-Baeza, Antonio Gonzalez, Zhenjiang Zech Xu, Alex Washburne, Hans H Herfarth, R Balfour Sartor, Rob Knight

**Affiliations:** 1 Department of Computer Science and Engineering, University of California, San Diego, California, USA; 2 Department of Pediatrics, University of California, San Diego, California, USA; 3 Department of Microbiology and Immunology, Montana State University System, Bozeman, Montana, USA; 4 Center for Gastrointestinal Biology and Disease, University of North Carolina, Chapel Hill, North Carolina, USA; 5 Division of Gastroenterology and Hepatology, Department of Medicine, University of North Carolina, Chapel Hill, North Carolina, USA; 6 Department of Microbiology and Immunology, University of North Carolina, Chapel Hill, North Carolina, USA

**Keywords:** intestinal microbiology, crohn’s disease

We read with interest the work by Pascal *et al* published recently in *Gut*.^1^ Here, they report the volatile microbial signatures of patients with Crohn’s disease (CD), a quality that greatly hinders our ability to classify healthy from affected subjects using 16S rRNA profiles from stool. Nonetheless, their work overcame these and other complications,^2^ producing a decision tree that classifies subjects with CD, UC, irritable bowel syndrome and anorexia. Although the authors note that both subtypes of IBD, particularly CD, have increased microbial community instability, this information is not used as a feature to improve classifier accuracy. Could microbiome instability become actionable by creating a new classifier that benefits from repeated measurements? If so, how many samples per individual are needed to assess instability?

We collected daily stool samples for up to 6 weeks from 19 CD subjects and 12 controls (see the analysis notebook for cohort description, methods and data, https://github.com/knightlab-analyses/longitudinal-ibd) over two separate periods of 2 or 4 weeks spread over 2 and 5 months, for a total of 960 samples. We believe that this is the most densely sampled longitudinal study of CD; previous studies collected samples every 1–3 months.^1 3^ Our cohort shows decreased alpha diversity and increased stability, as previously reported in CD and other subtypes of IBD.^1 3–5^ We also noted that subjects who underwent resection have lower alpha diversity than other CD-affected subjects (see analysis notebooks, https://github.com/knightlab-analyses/longitudinal-ibd).

**Figure 1:**
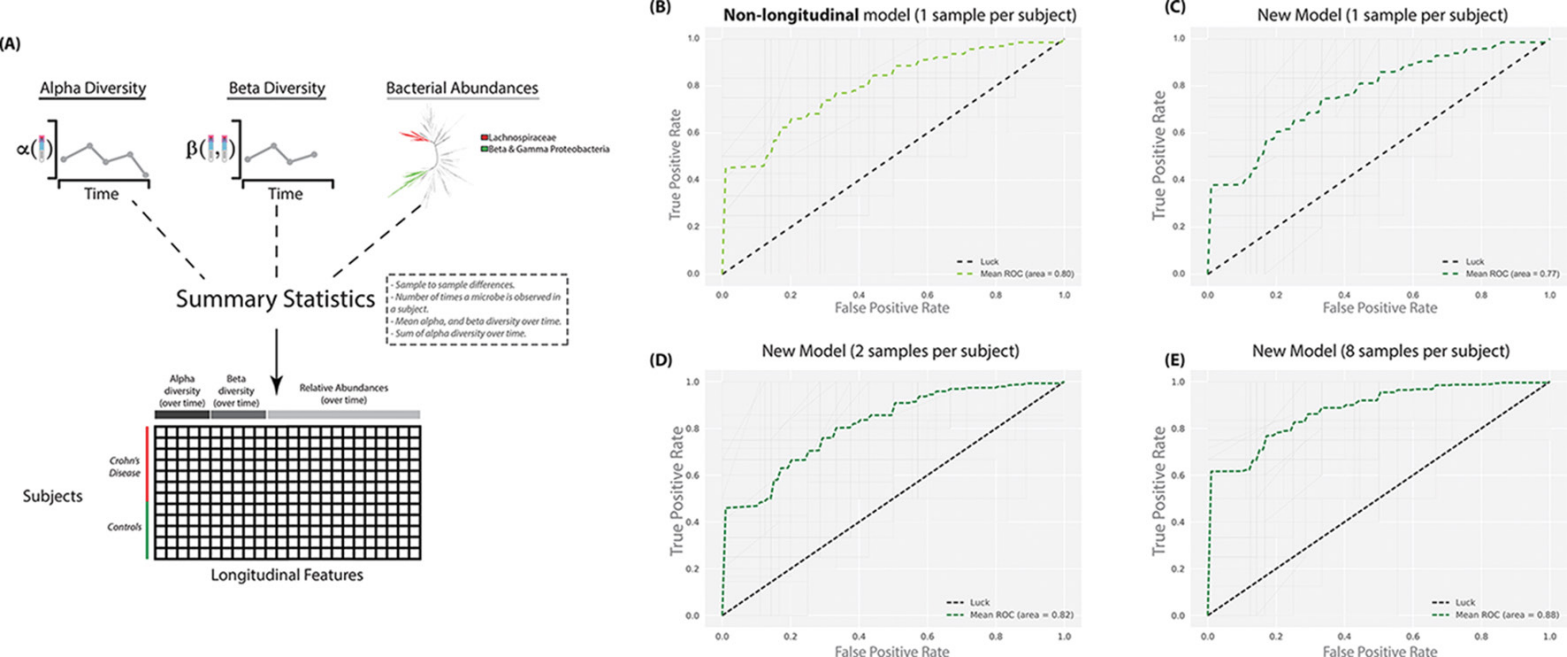
Diagram for the model creation and comparison of four receiver operating characteristic (ROC) curves. (A) Diagram describing the origin for the classifying features. (B) ROC curve for a model that relies on relative abundances and one sample per subject (as used in previous publications). (C–E) ROC curve for our new model at 1, 2 and 8 samples per subject. The grey lines represent the performance at each of the 100 iterations. The dotted black diagonal line represents the performance of a classifier that guesses the labels at random.

A critical experimental design question for clinical studies is whether a finite budget should best be spent collecting samples from more patients or collecting more serial samples from each patient? Therefore, we created a Random Forests^6^ model based on per subject aggregation of longitudinal data for alpha diversity,^7^ beta diversity^8^ and abundances of two phylogenetic factors found to be associated with CD in ileal biopsies^5 9^ (figure 1). With one sample per subject, our model performs worse than a classifier that uses microbial relative abundances at a single time point, but when more samples per subject are added, the classifier outperforms that approach and results previously only attained with biopsy samples.^5^ Furthermore, we replicate this observation with a different cohort (table 1).

**Table 1:** Performance summary of the classifier at increased samples per subject for this cohort (daily samples) and a previously published cohort

AUC	Samples per subject	Controls	Crohn’s disease	Sampling
0.80	1*	12	19	Daily samples
0.77	1	12	19	
0.82	2	12	19	
0.85	3	12	18	
0.86	4	12	18	
0.87	5	12	18	
0.87	6	12	18	
0.88	7	12	18	
0.88	8	12	18	
0.87	9	12	18	
0.87	10	12	17	
0.86	11	12	16	
0.80	1*	9	19	Monthly samples^3^
0.80	1	9	19	
0.83	2	9	15	
0.86	3	8	14	
0.92	4	8	12	

The AUC summarises the performance; closer to 1 is better, of the model trained on the different sample sizes as described by the other columns.*Represents the performance of a classifier that relies on non-longitudinal relative abundances only. AUC, area under the curve.

Novel analyses aggregating features over time and combining both alpha and beta diversity over time using our intensive daily sampling demonstrate that the main benefits are already obtained by collecting between three and five faecal specimens, and no additional benefits are obtained beyond seven serial samples. Similar results are found for monthly sampling. These results highlight the importance of treating CD as a volatile, time-varying condition, even during clinical remission, but provide hope to clinicians in that a relatively small number of samples yield large additional benefits, facilitating patient compliance. This information can be used to design collection of faecal samples for a large prospective cohort of patients with CD for longitudinal studies of host–microbial interactions over time.

The methods demonstrated here have not previously been used for microbiome analyses but have been used for other engineering applications, for example, in production lines to predict product specification outcomes in a steel manufacturer’s facility.^10^ We expect the results to generalise in other systems, including other GI and hepatic disorders, where dynamic features of the microbiome, host gene expression or other accessible descriptors can act as indicators of underlying dysbiotic states.
